# Colistin Induces Resistance through Biofilm Formation, via Increased *phoQ* Expression, in Avian Pathogenic *Escherichia coli*

**DOI:** 10.3390/pathogens10111525

**Published:** 2021-11-22

**Authors:** Na-Hye Park, Seung-Jin Lee, Eon-Bee Lee, Biruk Tesfaye Birhanu, Seung-Chun Park

**Affiliations:** 1 Laboratory Animal Center, Daegu Gyeongbuk Medical Innovation Foundation, Daegu 41061, Korea; pnh0211@dgmif.re.kr; 2 Reproductive and Developmental Toxicology Research Group, Korea Institute of Toxicology, Daejeon 34114, Korea; lee.seungjin@kitox.re.kr; 3 Laboratory of Veterinary Pharmacokinetics and Pharmacodynamics, College of Veterinary Medicine, Kyungpook National University, Daegu 41566, Korea; eonbee@gmail.com; 4 Cardiovascular Research Institute, Kyungpook National University, Daegu 41944, Korea

**Keywords:** antibacterial therapy, colistin, Avian Pathogenic *Escherichia coli* (APEC), antibiotic resistance, biofilm formation

## Abstract

This study aimed to optimize the colistin-based antibacterial therapy to prevent antimicrobial resistance related to biofilm formation in avian pathogenic *Escherichia coli* (APEC) in chicken. Of all the bacterial isolates (*n* = 136), 69 were identified as APEC by polymerase chain reaction (PCR). Through a series of antibiotic susceptibility tests, susceptibility to colistin (<2 μg/mL) was confirmed in all isolates. Hence, a mutant selection window (MSW) was determined to obtain colistin-induced resistant bacteria. The minimum inhibitory concentration (MIC) of colistin against the colistin-induced resistant APEC strains ranged from 8 to 16 μg/mL. To identify the inhibitory activity of colistin against the resistant strains, the mutant prevention concentration (MPC) was investigated for 72 h, and the single and multi-dose colistin activities were determined through the time-kill curve against APEC strains. Bacterial regrowth occurred after 12 h at a double MIC_50_ concentration (1.00 μg/mL), and regrowth was not inhibited even during multiple exposures. However, upon exposure to 8 μg/mL—a concentration that was close to the MPC—the growth of APEC was inhibited, including in the resistant strains. Additionally, colistin-induced resistant strains showed a slower growth compared with the susceptible ones. Colistin-induced resistant APEC strains did not show colistin resistance gene (*mcr-1*). However, the expression of higher *mgrB* and *phoQ* levels was observed in the resistant strains. Furthermore, these strains showed increased formation of biofilm. Hence, the present study indicated that colistin could induce resistance through the increased formation of biofilm in APEC strains by enhancing the expression of *phoQ*.

## 1. Introduction

Antibiotics are used to treat bacterial infections and to prevent further spreading of disease in humans and livestock. However, the emergence of antimicrobial resistance in several bacterial agents poses a threat for the future treatment and control of infections [1]. In particular, poultry farms, which are an important source of eggs and meat for human consumption, are alarmingly affected by drug resistant gram-negative bacteria. The indefinite application of antimicrobials in poultry farms is the main cause of the emergence of drug-resistant pathogens, such as *Escherichia coli* (*E. coli*) [2]. In poultry, the intestinal commensal avian pathogenic *E. coli* (APEC) may carry virulence genes to be turned pathogenic when the opportunity arises [3]. Specifically, APEC is a major pathogen of the Enterobacteriaceae family that causes colibacillosis, an acute and mostly systemic disease with respiratory symptoms [4,5,6,7].

To prevent APEC infections various antibiotics, including colistin, are used in poultry farms [8,9]. As colistin is effective against gram-negative bacteria—including those belonging to the Enterobacteriaceae family—it has been actively used in livestock production as a therapeutic drug or feed additive [10,11]. However, since the emergence of a transferable colistin resistance encoded by the *mcr-1* gene located on a conjugative plasmid in *E. coli* [12,13,14], the risk of colistin resistance development has been increasing alarmingly. In South Korea, from 2005 to 2015, 1.46% of strains isolated from livestock showed resistance to this antibiotic, and the percentage increased to 5.7% for strains isolated from infected chickens [8]. One of the risk factors for the occurrence of resistance is the administration of non-optimized drug dosage [15]. Several recent studies highlighted the importance of mutant prevention concentration (MPC)-based dosing approaches to enhance the potency of antibiotics and restrict the selection of resistant mutants by avoiding the traditional antibiotic treatment based on minimum inhibitory concentration (MIC) [16]. Antibiotic doses should be estimated considering drug concentration and treatment duration based on the MPC/MIC values, in order to not only target significant antimicrobial effects, but also to minimize the development of resistance. MPC is related to the mutant selection window (MSW), which is the antibiotic concentration range between the MIC and MPC. When an antibiotic concentration falls within the MSW, susceptible bacteria are inhibited as it exceeds the MIC; however, mutants are not inhibited as the concentration is below the MPC [16,17,18,19].

In addition, bacteria provide a survival mechanism by accumulating microbial aggregates on different surfaces [20]. Furthermore, sub-inhibitory antibiotic concentration also increases biofilm formation in bacterial isolates [21]. The biofilm formation in bacteria significantly contributes to antimicrobial resistance and makes infection treatment more difficult with a likely chance of relapse [22,23].

The drug administration interval and adequate doses are critical to ensure effective antibiotic treatment. In vitro studies of the time-kill assay, which simulates different antibiotic concentration-time profiles, offer a validated result to in vitro dynamic models and in vivo studies [17,24]. In particular, colistin is an antimicrobial agent that is not absorbed by the intestine [25]. Therefore, it is important to establish its dose through pharmacodynamic studies of the interaction between the drug and pathogenic strains, rather than through pharmacokinetics. Thus, in this study, we determined the effect of administration of sub-optimal dosage of colistin on colistin-induced resistance and its effect on the biofilm formation in APEC strains.

## 2. Results

### 2.1. Identification of APEC by PCR

**Figure 1:**
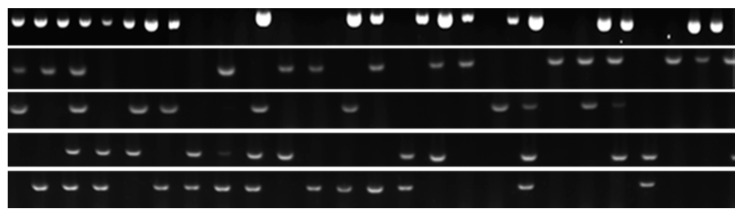
Identification of APEC strains isolated from infected chickens by hot-start PCR using *tsh* primer.

The expression of various genes of the APEC strains is presented in Table S1 and Figure S1. The *tsh* (L27423) gene was detected in 69 of the APECs, and the *E. coli* (ATCC 25922) and *Salmonella* Typhimurium (ATCC 14028) strains were used as negative controls (Figure 1). Furthermore, *fimAvMT78* (Z3750), *felA* (GCA_001620375.1), *fimH* (AJ225176.1), and *sta* (GPL3935) genes were only detected in APEC strains at a rate of 67%, 60%, 35%, and 30%, respectively. However, the expression of *yaiO* (EG13297), *fimA* (AF490890), and *iutA* (GCA_001021615.1) were identified in both the APEC and *E. coli* control strains. These strains were used for subsequent experiments on the pharmacodynamic profile of colistin against APEC variants (Table 1).

### 2.2. Antibacterial Activity of Colistin against APEC Isolates

The pharmacodynamic study indicated that the APEC clinical isolates had a MIC value between 0.0156 and 2 μg/mL, and they were sensitive to colistin (Table 2). The MIC_50_ and MIC_90_ measured 0.25 μg/mL and 0.5 μg/mL, respectively. The minimal bactericidal concentrations (MBC)/MIC ratio ranged between 1 and 2 (Table 3, Figure S2).

### 2.3. Antibacterial Susceptibility Profiles of APEC

The MIC values of the APEC strains were increased to 8–16 μg/mL after the MSW. The MPC of the susceptible strains was 32 μg/mL regardless of their MIC values, whereas that of the resistant strains was 128 μg/mL. The MBC/MIC ratio to all tested strains measured 1 (Table 3).

### 2.4. Antibiotic Concentration-Time Profiles of Colistin against APEC Strains

**Figure 2:**
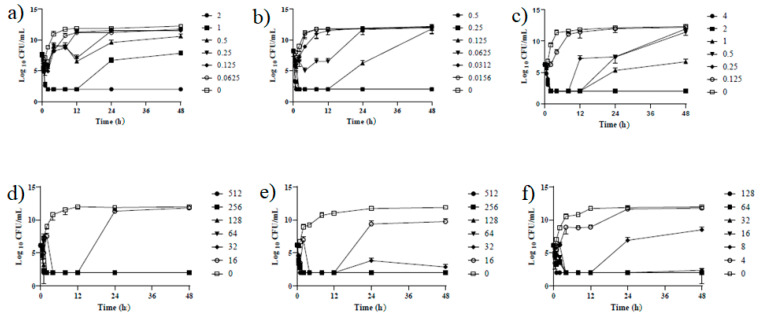
Time-kill curves of APEC strains. (**a**–**c**) represent the colistin-susceptible APEC strains; (**d**–**f**) represent the colistin-induced resistant APEC strains.

The duration of the antimicrobial activity of colistin against APEC strains was evaluated at a range of MIC concentrations (1–32 times of the MIC), including the previously mentioned breakpoint of colistin resistance (Figure 2, Table S3). Exposure to 2 μg/mL of colistin showed a 3-log reduction in the bacterial count within the first 2 h. However, at a concentration of less than 2 μg/mL, bacterial regrowth was observed after 8–12 h of incubation. In the resistant strains, regrowth was observed after 12 h of incubation at a concentration of less than 16 μg/mL, and the growth inhibition of resistant strains was confirmed near the value of the MPC. In addition, slower growth rates were shown in resistant strains compared with the susceptible ones.

### 2.5. Kill-Regrowth Analysis

**Figure 3:**
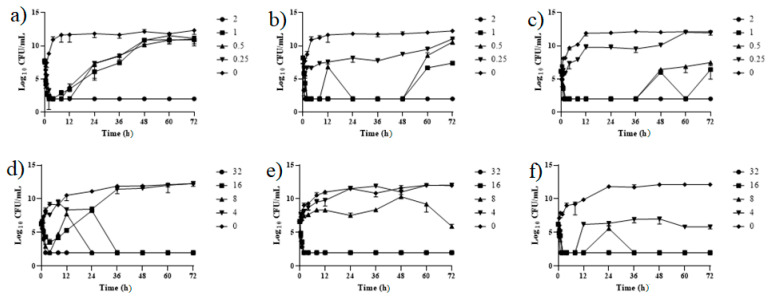
Time-kill curves of APEC strains after multiple exposures to colistin. (**a**–**c**) represent the colistin-susceptible strains, and (**d**–**f**) represent the resistant strains. The time kill assay was performed for 72 h by adding colistin every 12 h.

Based on the colistin concentration that induces bacterial regrowth obtained in the time-kill assay, further experiments were carried out to investigate whether repeated exposure to colistin at 12-h intervals would inhibit bacterial regrowth (Figure 3). In susceptible strains, the growth was temporarily inhibited after repeated doses of less than 1 μg/mL. However, regrowth was observed before 12 h, and the MIC value unevenly increased to 8 μg/mL after 72 h.

In the time-kill experiment, resistant strains showed regrowth after 12 h at a concentration of 16 μg/mL, but regrowth was inhibited when strains were exposed to colistin more than twice. In addition, bacterial growth was inhibited when the strains were treated three times with 8 μg/mL of colistin. However, a number of strains developed resistance and their MIC increased to 32 μg/mL. In some cases, due to multiple exposures to antibiotics, the resistant bacteria could not reach the maximum growth (10^11^ to 10^12^ CFU/mL) and remained in a static condition (Figure 3).

In addition, resistant bacteria characteristically showed a slower growth rate compared with the susceptible strains, with stationary phases lasting from 12 to 20 h, and from 8 to 12 h, respectively (Figure 2 and Figure 3).

### 2.6. Assessment of Biofilm Formation by Imaging System

**Figure 4:**
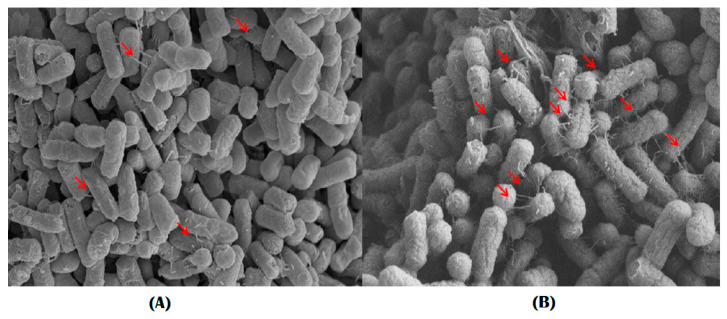
SEM image showing the presence of a putative pili in APEC cells. (**A**), a susceptible strain; (**B**), a resistant strain. Red arrows indicate the putative pili.

**Figure 5:**
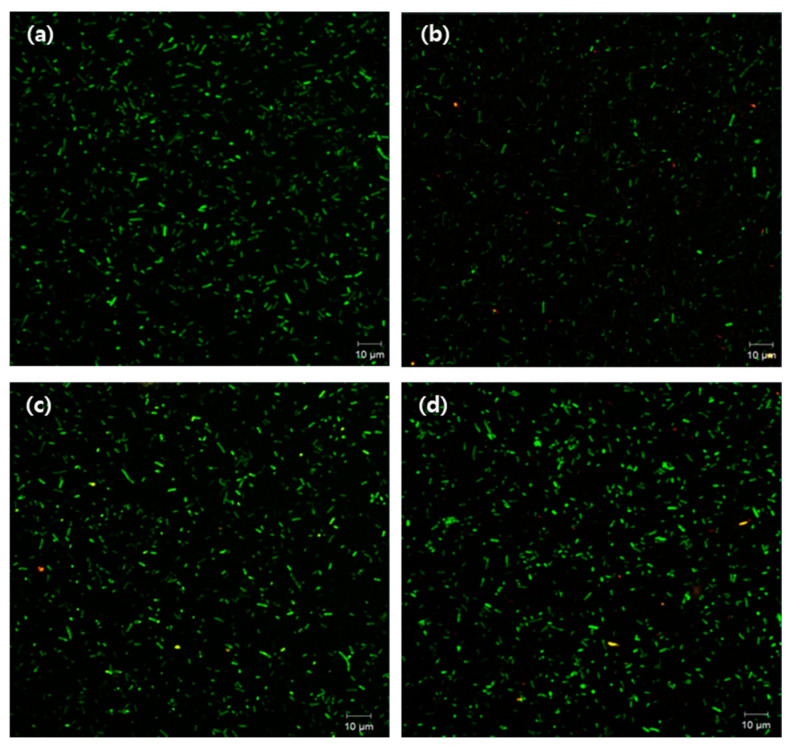
Confocal image showing live cells in biofilm forming APEC. (**a**) susceptible strain cultured in normal media, (**b**) susceptible strain exposed to colistin, (**c**) resistant strain cultured in normal media, and (**d**) resistant strain exposed to colistin.

The viability of biofilm in the presence of colistin was determined using scanning electron microscope (SEM) and confocal laser scanning microscopy (CLSM) techniques. Fimbria production increased in resistant strains (Figure 4), and CLSM results also showed that their cell viability was higher than that of susceptible strains after exposure to colistin (Figure 5). The results also indicated that, under suitable growth conditions, after 48 h biofilm formation was more pronounced in the colistin-resistant strains.

### 2.7. Analysis of Colistin Resistance Genes

**Figure 6:**
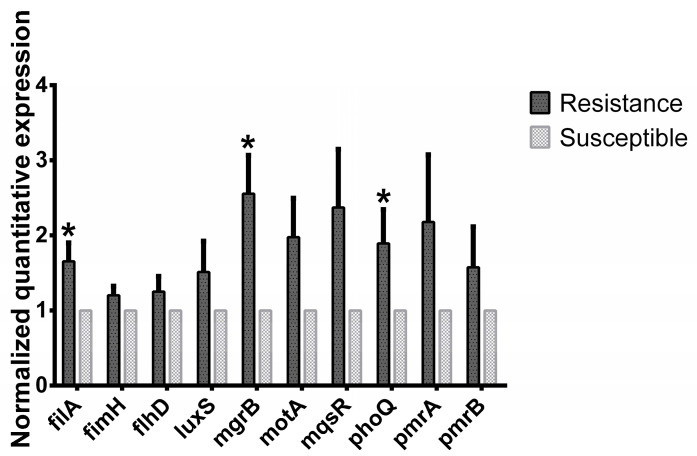
Relative virulence gene expression of susceptible and colistin-induced resistant APEC strains. Each virulence gene expression level was compared with the respective susceptible strain. * *p* < 0.05.

The results confirming resistance gene expression, obtained through PCR using *mcr-1* primers [11], showed that the *mcr-1* gene was not detected in all the isolates and colistin-induced resistant strains. However, the relative normalized gene expression level of *filA*, *mgrB*, and *phoQ* in the resistant strains increased significantly by 65.6, 155.9, and 89.3%, respectively. Furthermore, the genetic expression level of APEC genes—such as *fimH*, *flhD*, *luxS*, *motA*, *pmrA*, amd *pmrB*—also increased in the colistin-induced resistant strains. However, their level of expression was not significantly different from that of the susceptible strains (Figure 6).

## 3. Discussion

Colistin has been widely used in the poultry industry for decades as a therapeutic agent and growth promoter in several countries [26], mainly to prevent Enterobacteriaceae infections, including APEC. Until recently, colistin resistance was thought to present minimal risks due to the vertical transfer of mutations [23]. However, due to the emergence and spread of mobilized colistin resistance (*mcr*) genes in several countries, including South Korea, the use of this antibiotic has been restricted to treat enteric infections associated with *E. coli* [8,13,27,28]. Therefore, this study aimed to investigate the effect of colistin treatment on bacterial resistance development and biofilm formation in APEC strains using the MSW.

In this study, 136 clinical samples were obtained from the Gyeongsangbuk-do Veterinary Service Laboratory. A higher percentage of the APEC strains were identified by PCR using a temperature-sensitive hemagglutinin (*tsh*) gene, compared to the percentage reported previously [3]. In previous study, *tsh* gene was observed in about 70% of the APEC strains, which may indicate the possibility to identify these strains by PCR using this gene. Moreover, in our study, *tsh* was found in more than half of the soil samples, for a total of 69 APEC strains identified from 136 *E. coli* isolates obtained from poultry farm soils. This indicates the level of persistence of this pathogen, and the possibility of infection spreading through contaminated soil to the incoming healthy chickens, even after the infected ones are removed [29,30].

Antibacterial testing showed that the APEC strains isolated from the farms were susceptible to colistin. The MBC/MIC ratio obtained in this study is in line with previous studies [31,32]. However, the MPC of colistin-induced resistant strains was higher than that of various gram-negative bacteria reported in other studies regardless of the difference in MIC [33]. Moreover, bacterial regrowth was observed within 12 h after exposure to a higher colistin concentration in both susceptible and colistin-induced resistant strains. This suggests that repeated exposure to MIC-based colistin dosage can be a risk factor for the emergence of antibiotic-resistant bacteria. Cheng et al. also reported that exposure to colistin is a single factor which could lead to colistin resistance in *E. coli* [34].

The effect of repeated colistin exposure at various concentrations was determined to investigate whether the repeated administration of antibiotics at specific intervals would inhibit bacterial regrowth and resistance acquisition. The results showed that repeated exposure to colistin at less than 1.00 μg/mL at 12-h intervals did not inhibit bacterial regrowth in the colistin-susceptible strains. In addition, regrowth occurred even after repeating administration of the same concentration of colistin six times but increased the MIC of colistin against the APEC strain unevenly. The same pattern was observed upon repeated administration of enrofloxacin and marbofloxacin against *E. coli* [35]. This phenomenon increases the risk of selection of resistant mutants; therefore, it is suggested that the repeated administration of colistin at a concentration that is lower than its MPC value could lead to colistin resistance in APEC strains.

In contrast, the resistant strains were shown to regrow 12 h after treatment at the MIC value. However, the regrowth was inhibited after exposure to three times the MIC value. This suggests that antibacterial resistance can be prevented using MPC treatments during APEC infections. The MSW plays a significant role in optimizing dosage, improving the antibacterial effect, and reducing the occurrence of antimicrobial resistance by restricting resistant mutant selection [35,36].

In addition, resistant strains showed a slower growth rate compared with the susceptible ones, reaching a maximum growth of 10^12^ CFU/mL after 12 to 20 h of incubation, whereas the latter reached the stationary phase after 4–8 h. This could contribute to bacterial persistence and subsequent development of antimicrobial resistance, which may occur due to increased fitness costs [37,38].

Moreover, the expression of biofilm-forming and quorum-sensing genes was manifested by the slow growth rate of resistant bacteria in the colistin-resistant APEC strains—which is a characteristic phenomenon of biofilm-forming cells [39]—and increased biofilm formation was confirmed through image analysis by SEM and CLSM. The biofilm formation associated with APEC was accompanied by an increased number of pili in colistin-resistant strains compared with the susceptible ones. In addition, a higher proportion of living cells were observed in resistant APECs after exposure to colistin for 12 h, indicating a remarkably increased biofilm formation in these strains [40]. Bacterial regrowth is a prominent characteristic of biofilm-forming bacteria. Hence, regrowth observed in colistin-induced resistance in APEC strains could be related to biofilm formation [9,39,41].

The colistin-induced resistance in APEC strains did not show expression of the *mcr* gene. This is consistent with a previous study that indicated colistin-induced resistance could occur without regulation by the *mcr* gene [42]. The mcr-1 gene has been detected in 1% of wild isolates (10% of resistant strains) [8]. However, the presence of mutations on *mgrB* genes, which are the negative regulators, in the colistin-induced resistant strains could be responsible for their resistance [42,43,44]. Alteration of the *mgrB* gene is the predominant factor causing colistin resistance [42]. Furthermore, the increase in the expression of both biofilm-forming and quorum-sensing genes shown in this study could be associated with the alteration of *mgrB* in the resistant strains. This could be due to the dysfunctionality of the *phoPQ* two-component system, resulting from the mutation of *mgrB* [34,42]. Hence, *mgrB* mutations could be responsible for colistin-induced resistance by increasing the expression of biofilm-forming and quorum-sensing genes. Furthermore, *phoQ* senses environmental signals to activate *phoP* and contribute for colistin resistance [42,45,46,47]. The *phoPQ* system is critical regulating the expression of virulence genes in *Salmonella* spp. This signaling mechanism regulates biofilm formation in bacteria [48].

In conclusion, our results indicate that a MIC-dependent colistin concentration could not prevent the growth of APEC strains. On the contrary, it could induce the regrowth of bacteria, with the possibility of resulting in colistin resistance. Moreover, increased expression of the *phoQ* gene could lead to increased biofilm-formation in colistin-induced resistance APEC strains, which prevents colistin from reaching the site of action, finally inducing antibiotic resistance. Hence, colistin dosage should be optimized prior to infection treatment in poultry farms. However, further study to elucidate in detail the molecular mechanism allowing *phoQ* to regulate biofilm formation and confer resistance in APEC should be studied.

## 4. Materials and Methods

### 4.1. Isolation of Bacteria

Twenty APEC strains (*n* = 20) were acquired from the Gyeongsangbuk-do Veterinary Service Laboratory (GBVET), and isolates (*n* = 136) were obtained from infected chicken broilers and soil sampled in Korean poultry farms. Briefly, all isolates were cultured on MacConkey agar plates (BD, Sparks, MD, USA) at 37 °C, for 24 h. After incubation, APEC strains were identified by the presence of a temperature-sensitive hemagglutinin (*tsh*) gene, which is a conventional virulence factor in APEC [3], via hot-start polymerase chain reaction (PCR). A total of 89 strains were identified as APEC, including 20 strains acquired from the GBVET, and were used in the subsequent antibacterial susceptibility testing. Additionally, *E. coli* ATCC 25922 and *Salmonella enterica* subsp. enterica serovar Typhimurium ATCC 14028 were used as control strains. The resistant strains were grown in a medium containing a high colistin concentration. After MIC verification, three colistin-resistant strains were obtained, with MIC values ranging from 8 to 16 μg/mL [49].

### 4.2. Reagents

All the chemicals, reagents, and colistin sulfate salt were procured from Sigma-Aldrich (Sigma, St. Louis, MO, USA), and culture media were obtained from Becton Dickinson Biosciences (BD, Sparks, MD, USA).

### 4.3. Antimicrobial Susceptibility Assay

Antimicrobial susceptibility was performed using minimal inhibitory concentrations (MICs) and MBCs assays, based on the Clinical and Laboratory Standards Institute methods [49]. The pharmacodynamic profile of colistin against the APEC strains isolated from chicken and E. coli ATCC 25922 (quality control) were determined via in vitro studies. A total of 89 APECs at a concentration of 10^6^ CFU/mL were added into a 96-well microtiter plate containing two-fold, serially diluted colistin with a cation-adjusted Mueller Hinton Broth (MHB-II, BD, Sparks, MD, USA). The MIC was determined as the lowest concentration of the drug that showed invisible growth after incubation at 37 °C for 24 h. The MBC was determined by transferring 20 of the suspensions to a Luria-Bertani (LB) agar plate (BD, USA) starting from the MIC and cultured at 37 °C for 48 h.

### 4.4. Determination of the Mutant Prevention Concentration (MPC)

The resistant strains used in the experiment were obtained using the MSW. The colonies, which were grown on an agar plate containing a high concentration of colistin, were isolated, and the selected strains were examined. The MPC was obtained by spreading 10^10^ CFU/mL of the APEC strains over the MHB-II plate containing 1×, 2×, 4×, 8×, 16×, 32×, 64×, and 128× MICs of colistin. The plates were incubated at 37 °C for 72 h, and the MPC was determined as the lowest concentration that inhibited the growth of the APEC strains [13,33,50]. This assay was performed in triplicate.

### 4.5. In Vitro Pharmacodynamics by Time-Kill Assay

The time-kill assay was performed by inoculating 10^6^ CFU/mL of APEC strains in a tube containing 5 mL of MHB-II and 0, 1×, 2×, 4×, 8×, and 16× MICs of colistin for each strain, followed by an incubation phase in a shaking incubator for 48 h at 37 °C [49]. Samples were obtained at 0, 0.5, 1, 2, 4, 8, 12, 24, and 48 h after incubation, were serially diluted by 0.1% agar-saline, and were plated on LB agar plates and incubated at 37 °C for enumeration of viable colonies. Bactericidal activity was defined as a ≥3 log 10 reduction in bacterial counts (log 10 CFU/mL) from the original inoculum [51]. Time-kill curves were constructed by plotting mean colony counts versus time. The time-kill assay was performed in triplicate.

### 4.6. Killing and Regrowth by Multiple Exposures to Colistin

Based on the time-kill results, an experiment was designed to determine the inhibition, or acquisition, of antibiotic resistance after multiple exposures to colistin. Experimental conditions were the same as those mentioned above, except for the addition of colistin every 12 h after inoculation, and sample collection occurring at 12-h intervals for 72 h. The collected samples were serially diluted, and cultured on LB plates at 37 °C for 24 h. Additionally, the MIC values of samples were measured at each time point to confirm the acquisition of antibiotic resistance.

### 4.7. DNA Extraction and Primer Design for PCR

Bacterial DNA was isolated using the phenol-chloroform-isoamyl alcohol (PCI) method, as described previously [52]. Briefly, mid- to late-log phase culture bacteria (0.5 to 0.7 at OD_600_) were transferred to 1.7 mL e-tubes and centrifuged at 8000 rpm for 10 min. The pellet was dissolved in a 500 μL of extraction buffer and vortexed for 1 min. Then, 500 μL of PCI was added to each tube, and was mixed by vortexing for 1 min, and centrifuged at 12,000 rpm for 15 min. The supernatant was transferred to new 1.7 mL e-tubes, which were added with an equal volume of 100% ice-cold ethanol and mixed by inverting. After the last step of extraction was completed, DNA was precipitated with isopropanol at −80 °C for 20 min. Then, after centrifugation at 13,200 rpm for 10 min, the DNA was washed from the pellets with 70% ethanol and suspended in distilled water.

Primers were prepared as described in the listed references (Table S1), and they encoded the following adhesins: type 1 pili (*fim*), pili associated with pyelonephritis (*pap*), heat stable (*sta*) enterotoxins, temperature-sensitive hemagglutinin (*tsh*) [53,54]. Commercial PCR master mixes (AccuPower PCR PreMix, Bioneer, Daejeon, Korea) were used for the PCR reactions, which were carried out in 20-μL volumes at the amplification conditions described in Table S1. PCR products were visualized on a 1.5% agarose gel stained with ethidium bromide, at 100 V for 30 min.

### 4.8. Gene Expression Analysis

Genetic expression was performed using qRT-PCR. APEC strains was treated with a colistin sub-MIC for 16 h, and total RNA was extracted following the TRIZOL reagent protocol. The RNA purity was confirmed by measuring OD at 260 and 280 nm using a U-2800 spectrophotometer. cDNA was synthesized from 1 µg of RNA, using RNA to cDNA EcoDry Premix (Oligo dT), following the manufacturer’s instructions. Briefly, 1 uL of cDNA was added to the qRT-PCR mix including the primer set. A 20-μL PCR (AccuPower qPCR PreMix, Bioneer, Daejeon, Korea) reaction was carried out with the following amplification conditions: 95 °C for 3 min and 40 cycles of denaturation at 95 °C for 15 s, annealing temperature of 57 °C for 30 s. The primers for *mcr-1*, *mgrB*, *filA*, *fimH*, *motA*, *flhD*, *phoQ*, luxS, and *gapA* (as a house-keeping gene) genes were designed as previously described (Table S2) [44]. The gene expression levels were normalized to that of the *gapA* gene using the 2^−ΔΔCT^ method.

### 4.9. Comparison of Biofilm Formation by Imaging Analysis

Bacteria were cultured at 37 °C for 48 h in LB broth. At the end of incubation, the samples were centrifuged at 3000 rpm for 10 min. The obtained pellets were washed three times using phosphate-buffered saline (PBS, pH = 7.4) and fixed using 2.5% glutaraldehyde in PBS. After fixation, they were dehydrated with 30%, 50%, 70%, 90%, and 100% ethanol. Subsequently, samples were dried at a critical-point and coated with gold-palladium alloy. SEM imaging was performed with a FEI™ Nova NanoSEM 450 machine (FEI, Hillsboro, OR, USA).

Confocal microscopy was used to visualize and compare the difference between colistin-susceptible and resistant strains. Each strain was cultured on a chamber slide (Nunc^®^ Lab-Tek^®^ Chamber Slide™ system, Thermo Fisher Scientific, Waltham, MA, USA) at 37 °C for 48 h. The final density of the cultured bacteria was adjusted to an optical density of 1.0 (10^8^ CFU/mL) at 600 nm. After 48 h of incubation, each strain was treated with colistin at its respective MIC, and cultured again at 37 °C for 12 h. Strains were washed twice with PBS, and fixed overnight with 2.5% glutaraldehyde in 0.1 M PBS (pH 7.0) at 4 °C. The fixed samples were washed twice with PBS and were stained with LIVE/DEAD^®^ BacLightTM Bacterial Viability Kit (Molecular Probes, Burlington, ON, Canada) at 30 °C for 30 min. Samples were placed in two drops of 0.9% saline on the surface of a glass coverslip. Images were acquired within 30 min, using a Carl Zeiss (LSM700) confocal microscope [55,56].

### 4.10. Statistical Analysis

GraphPad Prism software (GraphPad Software, La Jolla, CA, USA) was used for statistical analyses, and the results were expressed as mean ± standard error of mean (S.E.M). Statistical significance was determined using the Holm-Sidak method with alpha = 5.000%.

## Figures and Tables

**Table 1:** Expression rate of the virulence factor in APEC strains.

Target Gene	APEC (%) ^1^	ATCC 25922 ^2^	ATCC 14028 ^3^
*tsh*	85	-	-
*fimAvMT78*	67	-	-
*felA*	60	-	-
*fim H*	35	-	-
*sta*	30	-	-
*yaiO*	90	+	-
*fimA*	100	+	-
*iutA*	60	+	-
*papC*	90	+	-
*papGIA2*	45	+	+
*papGJ96*	40	+	+

^1^ Avian pathogenic *Escherichia coli*, ^2^ *Escherichia coli* ATCC 25922, and ^3^ *Salmonella enterica* subsp. enterica serovar Typhimurium ATCC 14028 were used as control strains. + shows presence of the gene. Abbreviations: type 1 pili (*fim*), F11 fimbriae (*felA*), aerobactin presence (*iutA*), pili associated with pyelonephritis (*pap*), heat-stable enterotoxins (*sta*), temperature-sensitive hemagglutinin (*tsh*).

**Table 2:** MIC and MBC of colistin against APEC strains.

Potency	Colistin
Clinical isolates (*n*)	89
MIC (μg/mL)	
Range	0.0156–2
MIC_50_	0.25
MIC_90_	0.5
R (%) ^1^	0
MBC (μg/mL)	
Range	0.0156–2
MBC_50_	0.25
MBC_90_	0.5
MBC/MIC	1–2
ATCC 25922 ^2^	
MIC (μg/mL)	0.25
MBC (μg/mL)	0.25
MBC/MIC	1

^1^ R (%): Percentage of resistance in clinical isolates calculated using the breakpoint of colistin according to the CLSI, 2016 (susceptible, 2 μg/mL; intermediately resistant, 4 μg/mL; resistant, 8 μg/mL). ^2^ A control strain used.

**Table 3:** Antibacterial profiles of colistin against APEC strains.

Strain	MIC	MBC	MBC/MIC	MPC	MPC/MIC
S1 ^1^	0.06	0.06	1	32.00	512
S3	0.02	0.02	1	32.00	2048
S11	0.13	0.13	1	32.00	256
R1 ^2^	16.00	16.00	1	128.00	8
R3	16.00	16.00	1	128.00	8
R11	8.00	8.00	1	128.00	16

^1^ S denotes the colistin-susceptible avian pathogenic *Escherichia coli* (APEC) strains, and ^2^ R is the colistin-resistant APEC selected by MSW.

## Data Availability

All data generated for this study are contained within the article and Supplementary Materials.

## Supplementary Materials

The following are available online at https://www.mdpi.com/article/10.3390/pathogens10111525/s1. Figure S1: Identification of avian pathogenic *Escherichia coli* (APEC) by PCR, Figure S2: MIC distribution of isolated APECs, Table S1: Primer information, Table S2: Primers used in qRT-PCR analysis. Table S3: In vitro pharmacodynamic profile of isolates.
