# Supplementary material for: Colistin Induces Resistance through Biofilm Formation, via Increased phoQ Expression, in Avian Pathogenic Escherichia coli
Source: Pathogens. 2021 Nov 22;10(11):1525. doi: 10.3390/pathogens10111525 (PMC8620993; doi:10.3390/pathogens10111525)
Supplement: Supplementary file 1 [file pathogens-10-01525-s001.zip › pathogens-1440066-supplementary.pdf]

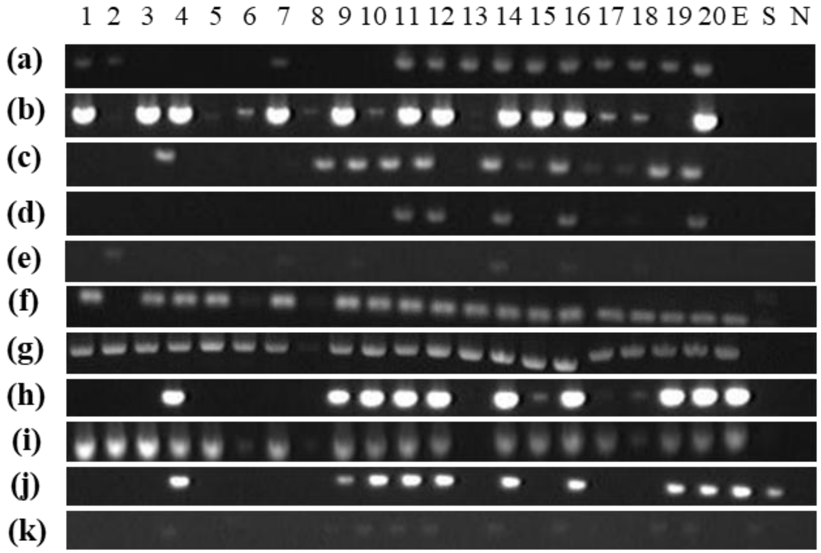

Figure S1. Identification of avian pathogenic *Escherichia coli* (APEC) strains by PCR. Lane (a) shows the expression of fimAvMT78, (b) is *tsh*, (c) is felA, (d) is *fimH*, (e) is *sta*, (f) is *yaiO*, (g) is *fimA*, (h) is *papC*, (i) is *iutA*, (j) is *papGIA2*, and (k) is *papGJ96*. Primer information is in Table S1. Horizontal numbers (1–20) mean each APEC strain, E means *E. coli* ATCC 25922, and S means *Salmonella enterica* subsp. enterica serovar Typhimurium ATCC 14028 and N means Negative control.

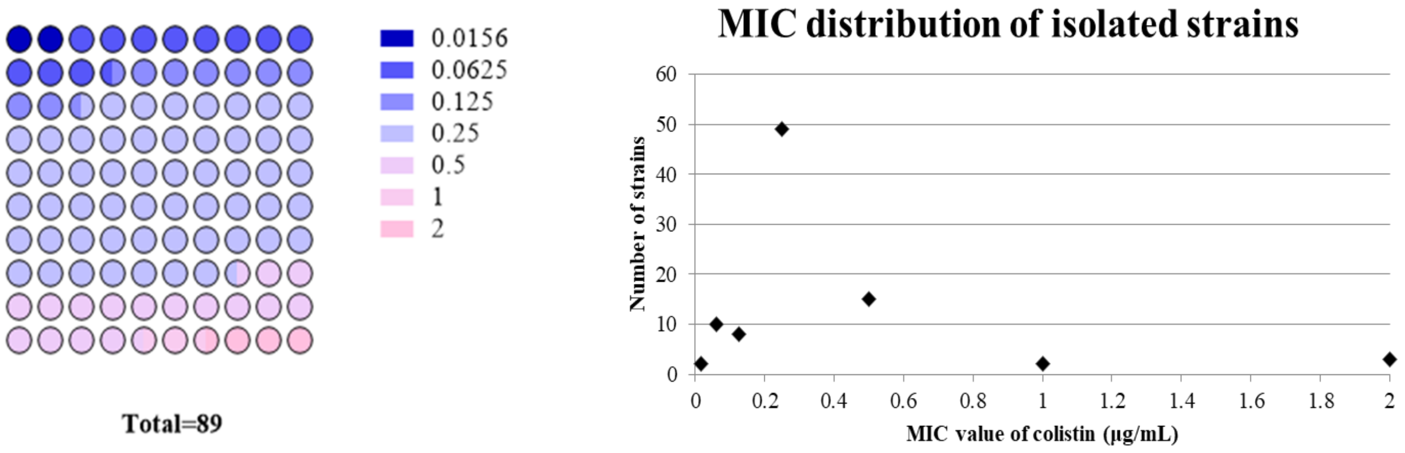

a **b**

Figure S2. MIC distribution of isolated APECs. MICs of 89 APECs were confirmed less than 2 µg/mL, that means colistin susceptible [49] (**a**). 49 APECs has 0.25 µg/mL MIC value, and it was determined MIC_{50} (**b**).

Table S1. Primer information.

Primer *	Primer Sequence	Size	Tm (°C)	Accession Number	Reference
APEC_fimAv$_{MT78}$_F	tctggctgatactacacc	266	52	Z37500	[38]
APEC_fimAv$_{MT78}$_R	actttaggatgagtactg				
APEC_tsh_F	ggtggtgcactggagtgg	640	58	L27423	[39]
APEC_tsh_R	agtccagcgtgatagtgg				
APEC_felA_F	ggtcaascagctaaaaacggtaagg	239	61	GCA_001620375.1	[40]
APEC_felA_R	ccttcagaaacagtaccgccattcg				
APEC_fim H_F	gatctttcgacgcaaatc	389	52	AJ225176.1	[41]
APEC_fim H_R	cgagcagaaacatcgcag				
APEC_sta_F	ttaatagcacccggtacaagcagg	147	55	GPL3935	[32]
APEC_sta_R	cttgactcttcaaaagagaaaattac				
*E. coli*_yaiO_ F	tgatttccgtgcgtctgaatg	115	58	EG13297	[42]
*E. coli*_yaiO_ R	atgctgccgtagcgtgtttc				
APEC_fimA_F	cggctctgtccctsagt	500	52	AF490890	[43]
APEC_fimA_R	gtcgcatccgcattagc				
APEC_papC_F	gacggctgtactgcagggtgtggcg	328	61	DQ010312.1	[44]
APEC_papC_R	atatcctttctgcagggatgcaata				
APEC_iutA_F	atgagcatatctccggacg	587	58	GCA_001021615.1	[33]
APEC_iutA_R	caggtcgaagaacatctgg				
APEC_papGIA2_F	gggatgagcgggcctttgat	190	63		[45]
APEC_papGIA2_R	cgggcccccaagtaactcg				
APEC_papGJ96_F	tcgtgctcaggtccggaattt	461	63		[46]
APEC_papGJ96_R	tggcatcccccaacattatcg				

* The primers are designed to target the following adhesins: type 1 pili (*fim*), F11 fimbriae (*felA*), aerobactin presence (*iutA*), pili associated with pyelonephritis (*pap*), heat stable enterotoxins (*sta*), temperature-sensitive hemagglutinin (*tsh*).

Table S2. Primers used in qRT-PCR gene expression.

Target gene	Sequence (5′–3′)
pmrA	F: acggtctgccttatgccatc
	R: tagcagataaatagcgtcagg
pmrB	F: atatccataacctgcgcgac
	R: gatgatattgaccacgagatt
mgrB	F: ttcatcacgcctgaatttac
	R: cgactcattccgaaaaagca
phoP	F: gggtaactcgacatgcaact
	R: tttcactttacctcccctcc
phoQ	F: aaagccgcgttttaacacca
	R: atgtactgatgggacgtctg
mcr-1	F: cggaattccaagatacaaattataaatactct
	R: acgcgtcgactcagcggatgaatgcggtgcggtc

Table S3. In vitro pharmacodynamic profile of isolates

	APEC 1	APEC 2	APEC 3	APEC 4	APEC 5	APEC 6	APEC 7	APEC 8	APEC 9	APEC 10
MIC	0.03–0.06	0.06–0.13	0.016	0.06–0.13	0.06	0.25	0.02–0.06	0.25–0.50	0.03–0.06	0.06–0.25
MBC	0.03–0.06	0.06–0.13	0.016	0.06–0.13	0.06	0.25	0.02–0.06	0.25–0.50	0.03–0.06	0.06–0.25
MBC/MIC	1	1	1	1	1	1	1	1	1	1
	**APEC 11**	**APEC 12**	**APEC 13**	**APEC 14**	**APEC 15**	**APEC 16**	**APEC 17**	**APEC 18**	**APEC 19**	**APEC 20**
MIC	0.06–0.13	0.0625	0.03–0.06	0.03–0.06	0.03–0.06	0.06–0.13	0.03–0.06	0.25	0.0156	0.06–0.13
MBC	0.06–0.13	0.0625	0.03–0.06	0.0625	0.03–0.06	0.06–0.13	0.0625	0.25	0.0156	0.125
MBC/MIC	1	1	1	1	1	1	1	1	1	1
	**APEC 21**	**APEC 22**	**APEC 23**	**APEC 24**	**APEC 25**	**APEC 26**	**APEC 27**	**APEC 28**	**APEC 29**	**APEC 30**
MIC	0.13	0.25	0.25	0.25	0.50	0.25	2.00	0.25	0.25	0.25
MBC	0.13	0.25	0.25	0.50	1.00	0.25	2.00	0.25	0.25	0.25
MBC/MIC	1	1	1	2	2	1	1	1	1	1
	**APEC 31**	**APEC 32**	**APEC 33**	**APEC 34**	**APEC 35**	**APEC 36**	**APEC 37**	**APEC 38**	**APEC 39**	**APEC 40**
MIC	0.25	0.25	0.50	0.25	0.25	0.25–1.00	0.25	0.25	0.25	0.25
MBC	0.25	0.25	0.50	0.25	0.25	2	0.25	0.25	0.25	0.25
MBC/MIC	1	1	1	1	1	2	1	1	1	1
	**APEC 41**	**APEC 42**	**APEC 43**	**APEC 44**	**APEC 45**	**APEC 46**	**APEC 47**	**APEC 48**	**APEC 49**	**APEC 50**
MIC	0.25	0.25	0.25	0.25	0.25	0.25	0.13	0.50	0.50	0.25

MBC	0.25	0.25	0.25	0.25	0.25	0.25	0.13	0.50	0.50	0.25
MBC/MIC	1	1	1	1	1	1	1	1	1	1
	**APEC 51**	**APEC 52**	**APEC 53**	**APEC 54**	**APEC 55**	**APEC 56**	**APEC 57**	**APEC 58**	**APEC 59**	**APEC 60**
MIC	0.25	0.13	0.25	0.25–0.50	0.13	0.25	0.25	0.25	0.25	0.25
MBC	0.25	0.13	0.25	0.50	0.13	0.25	0.25	0.25	0.25	0.25
MBC/MIC	1	1	1	1	1	1	1	1	1	1
	**APEC 61**	**APEC 62**	**APEC 63**	**APEC 64**	**APEC 65**	**APEC 66**	**APEC 67**	**APEC 68**	**APEC 69**	**APEC 70**
MIC	0.50	0.25	0.25–1.00	0.25	0.13–0.25	0.25	0.13–0.50	0.25–0.50	0.25	0.50
MBC	0.50	0.25	0.25–1.00	0.25	0.13–0.25	0.25	0.13–0.50	0.25–0.50	0.25	0.50
MBC/MIC	1	1	1	1	1	1	1	1	1	1
	**APEC 71**	**APEC 72**	**APEC 73**	**APEC 74**	**APEC 75**	**APEC 76**	**APEC 77**	**APEC 78**	**APEC 79**	**APEC 80**
MIC	0.50	0.25	0.13–0.25	0.25	0.25	0.25	0.25–0.50	0.50–2.00	0.13–0.25	0.50
MBC	0.50	0.25	0.13–0.25	0.25	0.25	0.25	0.25–0.50	0.50–2.00	0.13–0.25	0.50
MBC/MIC	1	1	1	1	1	1	1	1	1	1
	**APEC 81**	**APEC 82**	**APEC 83**	**APEC 84**	**APEC 85**	**APEC 86**	**APEC 87**	**APEC 88**	**APEC 89**	
MIC	0.50–2.00	0.13–0.25	0.25–0.50	0.25	0.13–0.25	0.25	0.25	0.25–0.50	0.25	
MBC	0.50–2.00	0.13–0.25	0.25–0.50	0.25	0.25	0.25	0.25	0.25–0.50	0.25	
MBC/MIC	1	1	1	1	2	1	1	1	1	
